# Clinical presentation of human monkeypox virus infection during the 2022 outbreak: descriptive case series from a large italian Research Hospital

**DOI:** 10.1186/s12985-023-02178-w

**Published:** 2023-09-18

**Authors:** Pierluigi Francesco Salvo, Damiano Farinacci, Francesca Lombardi, Arturo Ciccullo, Enrica Tamburrini, Rosaria Santangelo, Alberto Borghetti, Simona Di Giambenedetto

**Affiliations:** 1 Department of Safety and Bioethics, Section of Infectious Diseases, Catholic University of Sacred Heart, Rome, Italy; 2 Fondazione Policlinico Universitario A.Gemelli IRCCS, Infectious Diseases, Rome, Italy; 3 Infectious Diseases Unit, San Salvatore Hospital, L’Aquila, Italy; 4 Department of Basic Biotechnological, Clinical Intensivological and Perioperative Sciences, Catholic University of Sacred Heart, Rome, Italy; 5 Department of Infectology and Laboratory Sciences, Policlinico Universitario A.Gemelli IRCCS, Rome, Italy

## Abstract

**Background:**

In May 2022, a new case of Monkeypox Virus (MPX) was reported in a non-endemic area, the United Kingdom, and since then, the number of confirmed cases in Europe has been increasing until WHO, on May 10 2023, declared that MPOX is no longer a public health emergency of international concern. We aimed to describe the clinical and microbiological characteristics of sixteen patients with a confirmed diagnosis of MPX followed by a single Italian clinical centre, the Fondazione Policlinico Universitario Agostino Gemelli, between May 20 and August 30.

**Materials and methods:**

A prospective observational study has been conducted, collecting microbiological samples during the time of the infection, as well as epidemiological and clinical data of the patients. All patients provided written informed consent.

**Results:**

During clinical practice, 16 individuals presenting with consistent symptoms tested positive for MPX on a polymerase chain reaction. All patients were men having sex with men (MSM). The most frequent clinical presentation was a vesicular erythematous cutaneous rash, mainly distributed on the genital and perianal area, but also regarding limbs, face, neck, chest and back in some of the patients. Systemic symptoms, such as fever or lymphadenopathy, involved eight patients. The symptom most frequently reported by patients was pruritus in the area of the vesicles. Thirteen patients also reported pain. Nine patients were HIV-1 coinfected, but no significant differences have been observed compared to other cohort patients. The median time between the onset of symptoms and the healing was 19.5 days (IQR 14.0–20.3).

**Conclusions:**

Our cohort of patients presented a mild manifestation of the disease with no complications and no need for antiviral therapy nor hospitalization. This population seems different from the ones reported in the literature during the previous outbreaks in endemic areas in epidemiological data and clinical manifestations but also from a cohort of patients described in the literature from the 2022 outbreak, suggesting the importance for healthcare workers to keep in mind the possibility of an MPX infection in the differential diagnosis of patients presenting with consistent symptoms, even in non-endemic areas, to ensure efficient isolation of the patient for infection control purposes and effective management of the infection preventing the development of MPOX-related complications.

## Introduction

Human monkeypox is a rare zoonosis caused by Monkeypox Virus (MPXV), an Orthopoxvirus, similar in his clinical presentation to the smallpox infection, endemic in Central and Western Africa [1]. Two different clades of MPXV have been identified: a more virulent Congo Basin (Central African) and a less virulent West African clade [2].

At the beginning of May 2022, clusters of human monkeypox were identified in several non-endemic countries [3]. The first reported case of this new outbreak was reported in the United Kingdom on May 6th 2022, in an individual with a recent travel history to West Africa [4]. Since then, MPXV infection has been reported in an increasing number of countries worldwide in individuals without epidemiological links to endemic countries. Infected individuals during this outbreak appear to be mainly young men with unprotected sexual intercourses as the sole reported risk factor [5].

On July 23rd, WHO declared that the monkeypox outbreak represents a public health emergency of international concern. [3]

The first confirmed Monkeypox case in Italy was reported on May 20th, identified in a patient who recently travelled to Spain [6]. After that, the number of MPOX cases progressively increased, reaching a total of 957 confirmed cases throughout Italy, with a higher concentration in Lombardy (410 cases) and Lazio (161 cases) regions [7].

On May 10, 2023, the World Health Organization (WHO) declared that MPOX is no longer a public health emergency of international concern [8].

The decision was based on the consistent decline in global diagnoses. However, it was also emphasized that all countries worldwide should maintain their surveillance and response capabilities, integrating MPOX prevention and care into national health programs.

This study aims to describe the clinical presentation of monkeypox virus infection in individuals followed in the Fondazione Policlinico Universitario Agostino Gemelli IRCCS Hospital in Rome, Lazio, Italy.

## Methods

We collected clinical history and laboratory parameters of all patients with a consistent clinical presentation and a polymerase chain reaction (PCR) confirmed MPOX infection who came to our hospital between May 20th and August 30th 2022.

Clinical presentations drove microbiological sampling and laboratory testing; all patients had swabs collected from skin vesicles and pharynx, as well as urine and blood samples. These samples were also collected during follow-up visits. All collected samples underwent PCR testing for the research of the MPXV genome; the diagnosis of MPOX infection was confirmed in the case of positivity of at least 1 sample.

Patients with a confirmed diagnosis of human monkeypox took part in a video/telephone consultation to do counselling regarding the diagnosis and to assess follow-up.

For subjects co-infected with HIV, data regarding CD4 + cell count, HIV-RNA and CD4/CD8 ratio were also collected. All patients provided written informed consent for the storage and publication of anonymized clinical details and publication of clinical images.

## Results

During the study period, 26 patients presenting with consistent symptoms were tested for MPXV between May 20th and August 30th: 16 (61.5%) had positive PCR on at least one sample.

All confirmed monkeypox patients were men who identified themselves as MSM, with a median age of 41.5 years (IQR 33.75– 47.00). Four patients (25%) reported previous smallpox vaccination during childhood. All patients reported unprotected sexual intercourses prior to the appearance of the lesions or the onset of the symptoms. One of them came to our attention reporting previous sexual intercourse with a confirmed case of human monkeypox, showing disseminated vesicles and referring fever 48 h before the scheduled visit.

All patients showed up to be infected by the Western African clade (Clade II).

**Fig. 1:**
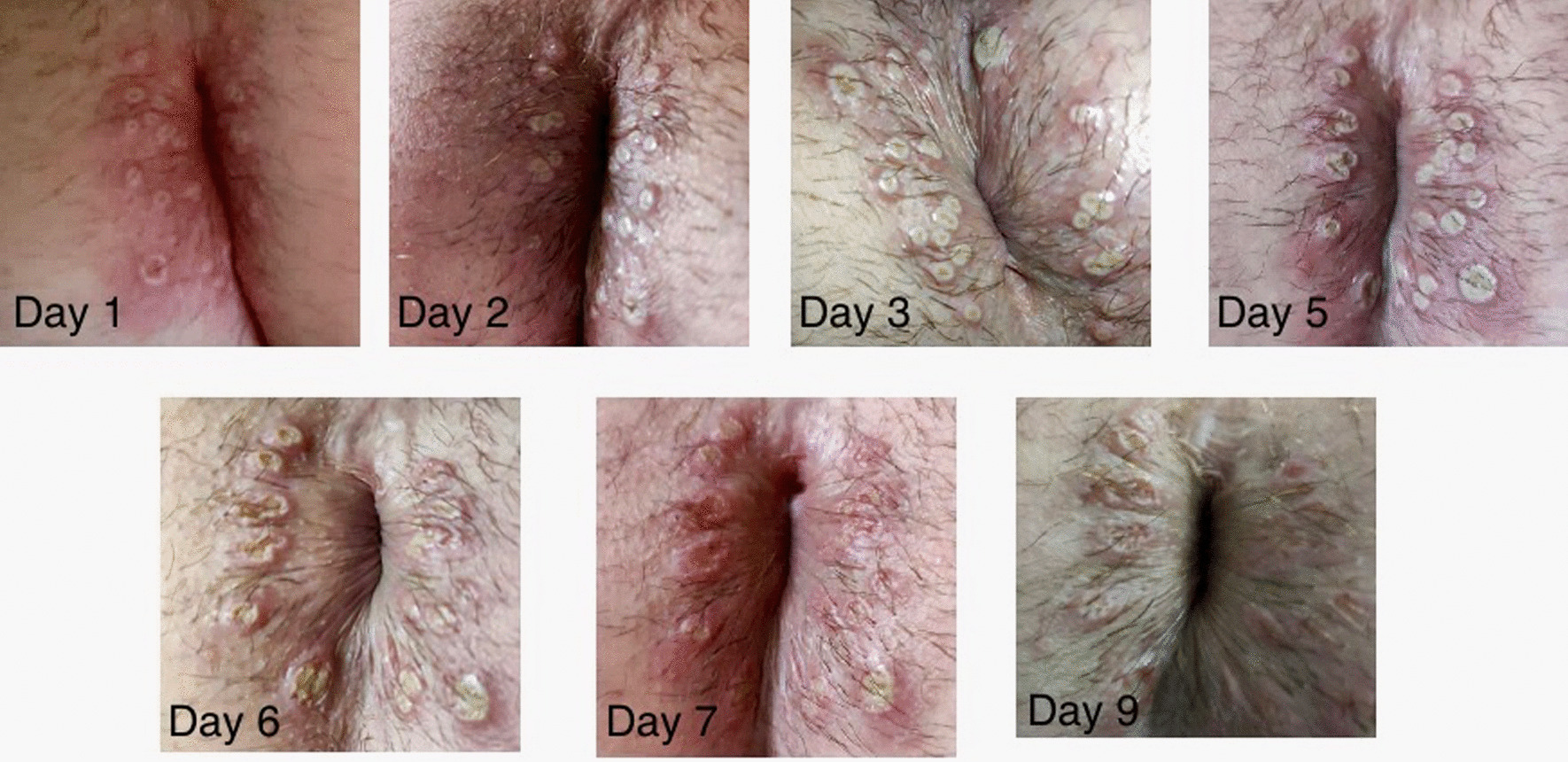
Clinical progression of skin lesions in the perianal area from day 1 (day of diagnosis) to day 9

**Fig. 2:**
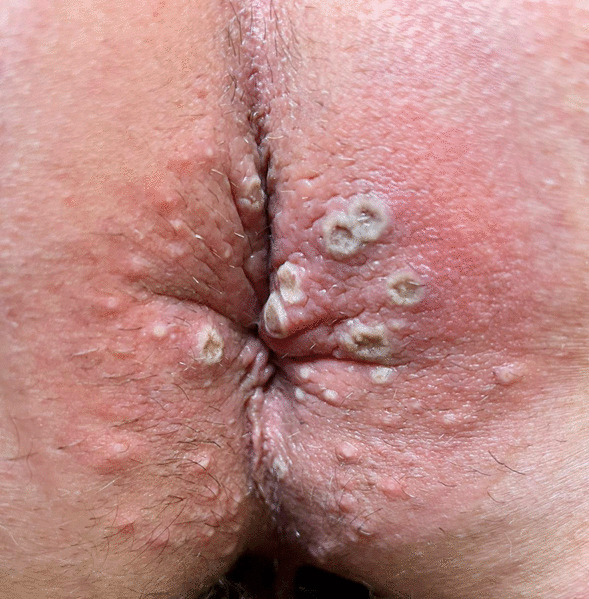
Cutaneous manifestation of MPOX infection in the perianal area of a PLWH

**Fig. 3:**
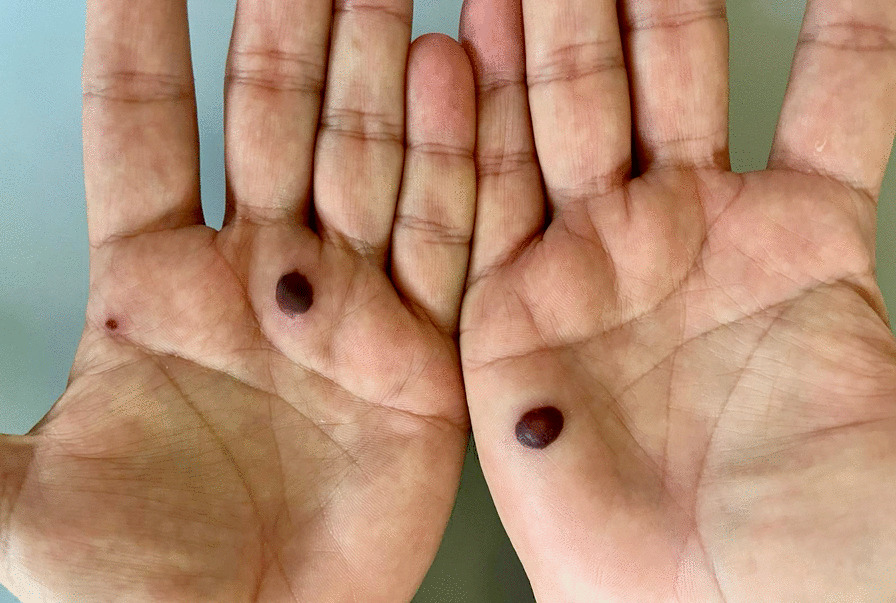
Bilateral cutaneous manifestations of MPOX infection

**Fig. 4:**
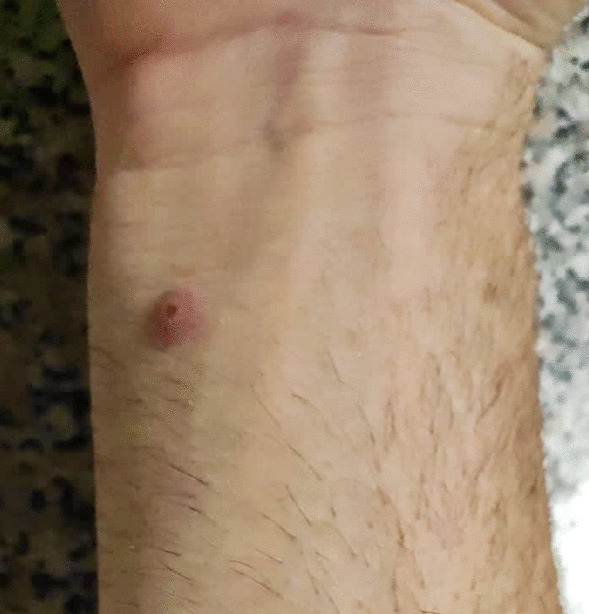
Skin lesion on the wrist of a person with MPOX infection

All patients who tested positive presented a cutaneous vesicular rash, more frequently on the genital area (N = 5, 31.25%) or the anus and the perianal area (N = 7, 43.75%). Nevertheless, vesicles have been found on the upper and lower limbs (N = 10, 62.50%) as well as on the back (N = 3, 18.75%), the face (N = 2, 12.50%), and the chest (N = 4, 25.00%) [Figs. 1, 2, 3 and 4]. Four patients did not present lesions on the genital or perianal area. Two patients (12.50%) only developed one vesicle (one on the penile shaft, one on the left hand); both had been vaccinated for smallpox during childhood. Eight patients reported systemic symptoms (50%), more frequent fever (N = 8, 50%) and lymphadenopathy (N = 6, 37.50%) in the inguinal area. None of these eight cases reported previous vaccination for smallpox. Systemic symptoms always appeared before skin lesions, with a median time between the two events of 1 day (IQR 0.00–2.0).

One patient reported a sore throat (6.2%) that spontaneously resolved in 3 days.

The most frequently reported symptom was pruritus, reported by 100% of patients, and pain localized on the site of the vesicles (N = 13; 81.25%), poorly responsive to pain relief therapy. In particular, patients relied on oral nonsteroidal anti-inflammatory drugs (NSAIDs) and paracetamol for pain management, ineffectively addressing the symptom. No patient in this cohort used analgesic drugs of a different class.

We observed anxiety and mood deflection in all patients described in this series, probably as a reaction to prolonged isolation but also as a consequence of the stigma regarding this disease.

Nine patients also had HIV-1 infection (56.25%); all of them were on highly active antiretroviral therapy (HAART) at the time of the diagnosis of MPXV infection and had a suppressed HIV-RNA (i.e. below 50 copies/mL). The median CD4 + cell count was 402 cells/mmc (IQR 325– 574) with a median CD4/CD8 ratio of 0.79 (IQR 0.61–1.17). No significant clinical presentation or outcome differences have been observed during our follow-up time for people living with HIV (PLWHIV). Three patients (18.75%) were on daily PrEP with Tenofovir disoproxil fumarate (TDF)/Emtricitabine (FTC). One patient had a resolved hepatitis B.

Regarding screening for concomitant STIs, one patient tested positive for Treponema pallidum (positive VDRL and TPHA) and required therapy after the resolution of the MPXV infection. Another patient tested positive for SARS-CoV-2 but did not develop symptoms nor require medical assistance or specific medication.

None of the patients had a severe form of the disease, and none of them required antiviral or antibiotic therapy nor hospitalization. No deaths were reported. The median duration of infection was 19.5 days (IQR 14.0-20.3).

All patients underwent follow-up visits 10 and 21 days from the beginning of the symptoms. Patients were considered healed when all of the following conditions were fulfilled: (a) clinical recovery, meaning the absence of systemic symptoms, (b) no new skin lesions appeared in the previous 48 h from the clinical evaluation, (c) negative polymerase chain reactions for the research of Monkeypox virus performed on blood and urine samples as well as skin and pharyngeal swabs.

All patients were isolated at home until the negativization of the microbiological tests for infection control purposes.

## Discussion

This case series analyzes the clinical characteristics of sixteen patients with human monkeypox infection diagnosed in a single Italian clinical center, aiming at describing clinical presentations and management of monkeypox virus infection in a non-endemic, high-income setting.

Overall, we observed a mild clinical presentation of monkeypox virus infection. Our patients only reported minor symptoms, with a complete clinical resolution after 7–10 days from diagnosis and no need for antiviral therapy.

Monkeypox cases in non-endemic areas are not new; several outbreaks have been reported in the past [9]. The most significant difference is the size and spread of the current outbreak compared to previous outbreaks, as well as the reported route of transmission.

The clinical features we observed in this cohort of patients are consistent with the characteristics described for the western African clade of monkeypox virus infection in the previous outbreaks, generally associated with a lower mortality rate [10]. The vesicles of the patients of this case series presented a similar natural history to those described in other similar case series [11] [12].

The clinical features described for our cases were comparable to the ones seen in Nigerian outbreaks of the western African clade of monkeypox virus in 2017-18 [13] and, in general, with the clinical manifestations described in the literature for the MPOX infection. Benites-Zapata et al., in a systematic review and meta-analysis comprising 19 articles published before June 2022, described the main clinical manifestations of MPOX infection [14]. Their work found that 25% of patients (95% CI 3–58%) experienced respiratory difficulties during the acute event, and 19% (95% CI 9–32%) had ocular involvement with conjunctivitis. Moreover, an overall hospitalization rate of 35% (95% CI 4–59%) was reported, with 4% of patients experiencing fatal outcomes due to complications.

Regarding the 2022 outbreak, the rate of hospitalization in non-endemic countries has been reported to be 11.1% in Portugal [15], 9.2% in the UK [16], 8.3% in Germany [17]. The reasons for hospitalization were related to pain management in cases that had become too severe or to managing complications.

In our experience, no patients exhibited respiratory symptoms or ocular involvement, and none required hospitalization, even for an HIV co-infection.

Furthermore, the patients described in this series present some differences from the ones described during previous outbreaks of the Monkeypox Virus Infection, in line with other findings in the literature describing cohorts of patients from the 2022 outbreak. First, all the patients are young men who referred to have had unprotected sexual intercourses prior to the beginning of the symptoms. All the patients had no epidemiological links to Western Africa. Most of the lesions are focused on the genital and perianal area, suggesting an association with sexual transmission.

The patients that reported a previous vaccination for the smallpox virus had a milder presentation of the infection, generally with one single papule and no systemic symptoms.

A potential limitation of our study is the relatively small size of our cohort when compared to other cohorts reported in the literature. Our outpatient clinic was not the designated referral center in Rome during the MPOX 2022 outbreak; nevertheless, we managed to observe a considerable number of MPOX infection cases, closely monitored with clinical and laboratory follow-up until the resolution of the acute condition.

Our study describes the clinical presentation of monkeypox virus infection in a real-life setting. What we consider most important is facilitating healthcare workers to provide an MPOX infection diagnosis in the shortest time possible, even in cases with few symptoms that could be more difficult to attribute to MPOX, especially in non-endemic countries. This would allow the patient to receive appropriate care during the acute phase, thereby preventing the onset of complications (e.g., superinfections, ocular or respiratory involvement) and hospitalization.

## Data Availability

Access to the database and data analyses were regulated by local institutional ethics committees and the Italian and European privacy legislation.
